# Passive Commuting and Higher Sedentary Time Is Associated with Vitamin D Deficiency in Adult and Older Women: Results from Chilean National Health Survey 2016–2017

**DOI:** 10.3390/nu11020300

**Published:** 2019-01-31

**Authors:** Patricio Solis-Urra, Carlos Cristi-Montero, Javier Romero-Parra, Juan Pablo Zavala-Crichton, Maria Jose Saez-Lara, Julio Plaza-Diaz

**Affiliations:** 1 PROFITH “PROmoting FITness and Health through Physical Activity” Research Group, Department of Physical Education and Sport, Faculty of Sport Sciences, University of Granada, 18071 Granada, Spain; 2 IRyS Research Group, School of Physical Education, Pontificia Universidad Católica de Valparaíso, Valparaiso 2374631, Chile; carlos.cristi.montero@gmail.com; 3 Departamento de Ciencias Farmacéuticas, Facultad de Ciencias, Universidad Católica del Norte, Avda. Angamos 0610, Antofagasta 1270709, Chile; javier.romero@ucn.cl; 4 Faculty of Education and Social Sciences, Universidad Andrés Bello, Viña del Mar 2531015, Chile; jzavala@unab.cl; 5 Department of Biochemistry and Molecular Biology I, School of Sciences, University of Granada, 18071 Granada, Spain; mjsaez@ugr.es; 6 Institute of Nutrition and Food Technology “José Mataix”, Center of Biomedical Research, University of Granada, Avda. del Conocimiento s/n. Armilla, 18016 Granada, Spain; jrplaza@ugr.es; 7 Department of Biochemistry and Molecular Biology II, School of Pharmacy, University of Granada, 18071 Granada, Spain; 8 Instituto de Investigación Biosanitaria IBS.GRANADA, Complejo Hospitalario Universitario de Granada, 18014 Granada, Spain

**Keywords:** Vitamin D, females, exercise, sedentary lifestyle, nutrition, elderly

## Abstract

The aim was to investigate the associations between different physical activity (PA) patterns and sedentary time (ST) with vitamin D deficiency (<12 ng/mL) in a large sample of Chilean women. In this cross-sectional study, the final sample included 1245 adult and 686 older women. The PA levels, mode of commuting, ST, and leisure-time PA were self-reported. Vitamin D deficiency was defined as <12 ng/mL and insufficiency as <20 ng/mL. A higher ST was associated with vitamin D deficiency (odds ratio (OR): 2.4, 95%: 1.6–4.3) in adults, and passive commuting was associated with vitamin D deficiency in older (OR: 1.7, 95%: 1.1–2.7). Additionally, we found a joint association in the high ST/passive commuting group in adults (OR: 2.8, 95%: 1.6–4.9) and older (OR: 2.8, 95%: 1.5–5.2) with vitamin D deficiency, in respect to low ST/active commuting. The PA levels and leisure-time PA were not associated with vitamin D deficiency. In conclusion, mode of commuting and ST seems important variables related to vitamin D deficiency. Promoting a healthy lifestyle appears important also for vitamin D levels in adult and older women. Further studies are needed to establish causality of this association and the effect of vitamin D deficiency in different diseases in this population.

## 1. Introduction

Vitamin D was first characterized as a vitamin in the 20th century and nowadays it is recognized as a prohormone [1]. Vitamin D has two major forms, vitamin D_2_ (ergocalciferol) and vitamin D_3_ (cholecalciferol). The first is derived from plant sources, is not largely human-made, and added to foods, and the latter is synthesized in human skin and is consumed in diets via animal-based foods intake, mainly fish oils [1]. The active form of vitamin D generates a number of extraskeletal biological responses including inhibition of breast, colon, and prostate cancer cell progression; effects on the cardiovascular system; and protection against a number of autoimmune diseases including multiple sclerosis and inflammatory bowel disease [2]. 

Vitamin D is important in biological process related with women’s health, such as fertility, pregnancy outcomes, and lactation at young age [3]. Some observational studies have revealed that a decrease in the vitamin D levels in women is related with reduced fertility [4], antenatal and postpartum depression [3]; as well high parathyroid hormone, the increasing risk of suffering sarcopenia and impaired glucose metabolism in the general population [5,6]. Moreover, supplementation with vitamin D probably might reduce the rate of falls but not risk of falling in older people [4,7]. Normal levels of vitamin D are associated to a low frequency of pathogenesis and neoplasm progressions, as well as hypertension, diabetes, immune disorders such as multiple sclerosis, musculoskeletal conditions [8,9], or bone mass status, especially in women [10]. Although vitamin D deficiency is rare in developed countries, subclinical forms occur, and they have public health relevance since low vitamin D concentrations are highly prevalent among population living in high latitudes, mainly indoors, and among those who are older or dark skinned [11]. Sunlight is the primary source of vitamin D [12], a very rough general estimation indicates that about 80% of vitamin D supply comes from skin ultraviolet-B-induced production, whereas only 20% comes from dietary intake; however, this varies considerably depending on different factors such as seasonal/sun exposure habits, latitude, nutrition/supplement intake or ethnicity [13]. 

In 2013, a review identified 243 studies related with vitamin D status in Caribbean and Latin American countries. The final analysis only included 28 studies with two major characteristics: small samples sizes and low national representativeness. Countries with available data have shown that vitamin D insufficiency was classified in the range of mild, moderate, or severe public health problem, and the only country with a nationally representative sample in this study was Mexico [14]. In Chile, a study conducted in 2007 with 555 post-menopausal women showed a vitamin D deficiency in 47.5% of the population, defined as less than 17 ng/mL [15]. Recommendations of normal levels of vitamin D in plasma are above of 30 ng/mL, and different cut-points have been used to classify insufficiency or deficiency, according the population [16,17]. For instance, recent data from the Chilean population showed that 80% of older subjects with a low-energy hip fracture have presented insufficient vitamin D levels (less than 20 ng/mL) [18]. Moreover, in healthy older populations, vitamin D values were very similar, 83% of women and 55.3% of men presented parameters below 20 ng/mL [19].

Regular physical activity (PA) and low sedentary time (ST) have significant benefits for health at all ages [20]. The principal recommendation is some PA is better than doing none [21]. The adult population (aged from 18–64 years) and older population (aged 65 years and above) should do 150 minutes of moderate-intensity PA throughout the week or at least 75 minutes of vigorous-intensity PA throughout the week, or an equivalent combination of moderate- and vigorous-intensity activity (e.g., walking, cycling, or doing sports) [20]. Globally, around 23% of adults aged from 18 and above, were not active enough in 2010 (men 20% and women 27%) [4,7,22,23,24,25]. Physical activity increase may be about general PA level, leisure PA, mode of commuting, organized sports, among others and independently reducing ST [20].

Previously, low serum vitamin D levels have been associated with lower PA level, gait speed, and balance. Vitamin D level below 30 ng/mL was not associated to an increased risk of fractures; however, a subgroup of women with serum vitamin D levels below 20 ng/mL showed an increased risk tendency of fractures, which may be associated to an inferior PA and postural stability [26]. Another report demonstrates that serum vitamin D concentrations below 20 ng/mL are associated to a poor physical performance, that is to say to a decreased physical performance in older men and women [27]. On the other hand, evidence shows that cycling is associated to high UV exposure, and thus, to high serum vitamin D levels compared to most other outdoors activities that are practiced, including walk [28,29]. It has been established that not all activities are equivalent regarding to sunlight exposure. Thus, whereas some patterns of PA have been associated with vitamin D levels, there is a lack of studies considering ST of mode of commuting. Interestingly, it is necessary to establish a relationship of the aforementioned according to the different population, since it has been shown that older people would be at risk [30]. Furthermore, a better understanding of the effects, according to the population, could help to provide valuable lifestyle recommendation.

Finally, data from general Chilean population regarding vitamin D levels are scarce, and associations with PA patterns and ST might be important factors in the status of vitamin D and maintenance of health [22]. Therefore, the aim of the present study was to investigate associations between self-reported PA patterns, ST, and different serum vitamin D levels in a large sample of Chilean adults and older women, adjusting for a number of potential confounders.

## 2. Material and Methods

### 2.1. Study Population

The 2016–2017 Chilean National Health Survey was a representative household survey with a stratified multistage probability sample of 6233 non-institutionalized participants over 14 years old from the 15 regions in Chile, both urban and rural. This survey represents the first, largest, and representative measurement of serum vitamin D levels in Chilean people. Sample size was calculated with a relative sampling error of less than 30%, and absolute sampling error of 2.6% to national level. The participation rate was 90.2%. Detailed information about the survey has been described elsewhere [31]. Vitamin D measurement was taken from a subsample of fertile age women (15 to 49 years) and older people. In this cross-sectional study were included those who have complete vitamin D measurement, valid response in PA questions and correct anthropometric parameters. Final sample was divided in adults (≥18 to <65 years) and older (≥65 years) groups, both for Chilean women. The ethics committee of the Pontificia Universidad Católica de Chile and the Chilean Ministry of Health approved the study protocol and ethical consent forms. 

### 2.2. Survey and Sample

Standardized protocols were used and all investigators (nurses and research technicians) underwent joint training sessions prior to implementation of the survey. The fieldwork for this survey was conducted between August 2016 and March 2017; while blood samples and laboratory tests were made between September 2016 and February 2017.

### 2.3. Serum Vitamin D Levels 

A nurse took venous blood samples in morning hours. Serum was extracted from 1 mL of total blood. Standardized liquid chromatography-tandem mass spectrometry (LC–MS/MS) method was used for measurement of 25(OH)-Vitamin D_3_ for Chilean National Health Survey 2016–2017, which allows laboratories and surveys to compare 25(OH)-Vitamin D_3_ measurements [32]. Vitamin D levels were categorized according two different criteria: (i) specific criteria for Chilean population according to the Health Ministry of Chile [33] of deficiency as <12 ng/mL [34], and (ii) internationally frequent cut-points used criteria corresponding to insufficiency of <20 ng/mL) [16,17]. This method has better analytical specificity and sensitivity compared to immunoassay methods, and fixed analytical goals for imprecision (≤10%) and bias (≤5%) [35].

### 2.4. Physical Activity

The Global PA Questionnaire (GPAQ) (version 2) to measure PA was used. The physical active categories were defined according to standard criteria of the questionnaire. Those who had less of 600 metabolic equivalent of task (METS) per week were considered inactive and those who had 600 or more METS per week were considered active [36]. 

### 2.5. Leisure-Time Physical Activity

A question was made in the visit, the question was (i) *In the last month, Did you practice sport or did any PA out of work time, during 30 minutes or more each time*? The response options were: (i) Yes, three times a week or more; (ii) Yes, one or two times a week; (iii) Yes, less of four times per month; (iv) I do not practice sport. The responses then were categorized in “Yes” for those who exercise three times a week or more, and “no” for those who did not practice sport at least three times a week.

### 2.6. Commute Mode

A question was made in order to inquire the commute mode of every surveyed, (i) which is the mode of commuting that you use (at least one time per week?) The response options were: (i) drive a light car; (ii) drive a heavy car; (iii) light car passenger; (iv) heavy car passenger; (v) bicycle; (vi) walk; (vii) and other. The responses were categorized in “active commuting” for those had mode of commuting bicycle or walk, and “passive commuting” for the rest.

### 2.7. Sedentary Time

A question of the GPAQ to estimate ST was asked to every participant of the study. The question was (i) *How much time do you usually spend sitting or reclining on a typical day*? The participant had to respond in minutes and hours per day. This question was categorized according to low ST (<4 hours per day); middle ST (≥4 and <8 hours per day); and high ST (>8 hours per day) [37].

### 2.8. Covariates

Socio-demographic data were collected for all participants, including age (years), menopausal status (yes/no), achieved education level (primary/secondary/beyond secondary), region (I to XV) and dairy consumption (three times a day or less, once each day, each two days, once a week, once a month or definitely never). Further, participants were asked according their sunlight exposure during the last week, (i) How much sunlight have you been exposed to in the last week? The responses were: (i) much; (ii) little.

### 2.9. Statistical Analysis

**Figure 1:**
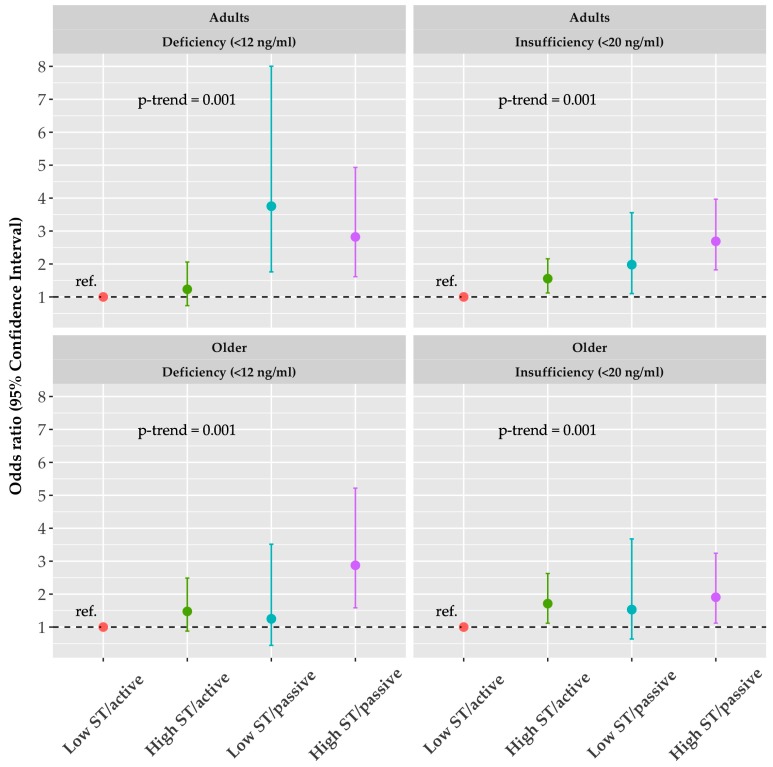
Odds ratio of the joint association between sedentary time (ST) and mode of commuting with different cut-points of vitamin D deficiency in adults and older women. The reference categories are groups with low ST (<4 hours/day) in combination with active commuting.

Data were presented as mean, standard deviation (SD), and percentages (%). Independent *t*-test and chi-square test were used to compare differences between adults and older women for continuous and categorical variables, respectively. Firstly, separated multivariable logistic regression model were employed to obtain odds ratio (OR) and confidence interval (CI 95%) in respect to different cut-points, adjusted by age (years), menopausal status (yes/no), achieved education level (primary/secondary/beyond secondary), region (I to XV), dairy consumption (three times a day or less, once each day, each two days, once a week, once a month or definitely never), and sunlight exposure (much/little). Finally, joint associations of ST and commute mode according to different criteria were tested. Here, ST was categorized as low ST (<4 hours per day) and high ST (≥4 hours per day) and it was combined with active and passive commuting. Thus, low ST/active commuting was used as reference group, high ST/active commuting, low ST/passive commuting, high ST/active commuting were second, third, and fourth groups, respectively. The performed model was adjusted by the same covariates mentioned previously plus PA level (active/inactive). For the interpretation of odds ratio, the effect size cut points of 1.68, 3.47, and 6.71 were used, according to small, medium, and large effect size [38]. Analyses were performed using SPSS-IBM (Software, v.21.0 SPSS Inc., Chicago, IL, USA), and a value of *p* < 0.05 was considered statistically significant. The Figure 1 was performed using the ggplot2 package in R and Supplementary Materials Figure S1 with leaflet package.

Figure 1 (bottom panels) shows joint OR for vitamin D deficiency for older women according the sedentary group and mode of commuting group. It can be appreciated from Figure 1 that for the cut-points of <12 ng/mL, compared to the reference group, the high ST/active group presents an OR of 1.476 (CI: 0.877–2.487, *p* = 0.143) the low ST/passive group presents an OR of 1.25 (CI: 0.444–3.514, *p* = 0.673) and high ST/passive group presents an OR of 2.875 (CI: 1.584–5.218, *p* = 0.001) for vitamin D deficiency. Furthermore, according to cut-points of <20 ng/mL, it can be appreciated in Figure 1 an OR of 1.712 9CI: 1.116–2.627, *p*: 0.014) for high ST/active, OR of 1.531 (CI: 0.638–3.676, *p* = 0.34) low ST/passive and OR of 1.905 (CI = 1.119–3.242, *p* = 0.018) high ST/active groups.

## 3. Results

The present study included only those who had a complete vitamin D measurement, valid responses in PA questions, and correct anthropometric measurements. The sample with serum vitamin D measurement was 2326. The final sample was divided into adults (≥18 years) and older (≥65 years) Chilean women groups. The final sample with complete data of physical activity patterns and covariates included was 1931 women. Table 1 shows descriptive characteristics of participants separated by group of age. 

The anthropometric and nutritional values show similar results in both groups. The distribution of underweight, normal weight, overweight, and obese was the same for adult and older women. Vitamin D levels expressed as ng/mL were 20.2 for adult women and 18.0 for older women. Adult women exhibited higher vitamin D levels compared with older women, as well as higher PA patterns, leisure-time PA, educational level, and sunlight exposure. In contrast, ST, dairy consumption and menopausal status were lower in adult women compared to older population. Finally, the commute mode was similar in both groups. Table 2 shows the results of logistic regression analysis for each PA pattern according different cut-points and separated by adults and older women. 

### 3.1. Adults

Table 2 (left side) shows the OR of vitamin D deficiency for adults. It can be seen that middle and high ST groups are associated with vitamin D deficiency (OR between 1.7 to 2.6, all *p* < 0.001) in both cut-points. Furthermore, Table 2 shows that passive commuting are also associated with vitamin D deficiency only for cut-points less than 20 ng/mL. PA level pattern and leisure-time PA pattern were not associated with vitamin D deficiency (all *p* > 0.05).

Figure 1 was constructed using two variables, ST and commute mode. These variables were categorized as low and high, and passive or active, respectively. The OR of the joint association between ST and mode of commuting was determined with different cut-points of vitamin D deficiency in adult women (Figure 1, upper panels) and older women (Figure 1, bottom panels). Reference categories were groups with low ST (<4 hours/day) in combination with active commuting. It can be appreciated from Figure 1 that for the cut-points of <12 ng/mL, compare to reference group, the high ST/active group presents OR of 1.231 (CI: 0.735–2.060, *p* = 0.43), the low ST/passive group presents OR of 3.754 (CI: 1.761–8.000, *p* = 0.001) and high ST/passive group presents OR of 2.821 (CI: 1.614–4.931, *p* = <0.001) for vitamin D deficiency. Furthermore, according to cut-points of <20 ng/mL, it can be appreciated an OR of 1.555 (CI: 1.120–2.158, *p* = 0.008), OR of 1.980 (CI: 1.102–3.558, *p* = 0.022) and OR of 2.689 (CI: 1.822–3.967, *p* = <0.001), for high ST/active, low ST/passive and high ST/active groups, respectively.

### 3.2. Older

Table 2 (right side) shows the OR of vitamin D deficiency for older women. Middle ST group shows a high vitamin D deficiency and high ST group showed a tendency association (*p* = 0.004 and 0,074, respectively) only in <12 ng/mL cut-points. Passive commuting is associated with vitamin D deficiency in both cut-points. PA level group and leisure-time PA group were not associated with vitamin D deficiency (all *p* > 0.005).

## 4. Discussion

We examined the association between PA patterns and ST with vitamin D deficiency and insufficiency in a nationally representative sample of Chilean women. The results showed that passive commuting is associated with vitamin D deficiency and insufficiency in older women, whereas high ST is associated with vitamin D deficiency and insufficiency in adult women. Additionally, we identify a joint effect of high ST/passive commuting on vitamin D deficiency and insufficiency in both groups. The magnitude of the effect was between small to medium.

To our knowledge, this is the first study that analyzes how different patterns of PA and ST are related with vitamin D levels in Chilean population. Interestingly, active adult women and inactive older women shares the same value, as well as inactive adult women correspond to active older women, creating a mirror effect. The vitamin D levels expressed as ng/mL was 20.2 for adult women and 18.0 for older women. These results were similar to another study in a little sample of Chilean older people (*n* = 57 participants); the aforementioned study have reported that women presented lower levels than men 15.6 ± 5.8 and 19.2 ± 6.0 ng/mL, respectively.

In Europe, vitamin D insufficiency (<20 ng/mL) is present in 36.0% of younger and 24.4% of older participants [39], while there is a high variability between countries. Contrary, to our results, the prevalence of vitamin D insufficiency was higher in young people compared to older participants; an explanation given by the authors is that older population are healthier and quite more active than younger participants. These differences are connected, in some cases, to institutionalization factors, especially combined with concurrent health and mobility problems, such as reduced skin efficiency to produce endogenous vitamin D levels [40], poor dietary vitamin D intake as well poor general nutritional status [41]. Thus, the evidence is not conclusive regarding vitamin D levels according to age, since other risk factors have been identified as skin pigmentation, latitude, health status, vitamin D intake by fortified food or any behavior related to sunlight exposure such as the use of lighter-weight clothes, or indoor working [42,43,44].

Physical Activity has been proposed to be an important determinant of vitamin D status in Caucasian adults. Jerome et al. [45] showed that sedentary students possessed significantly lower vitamin D levels compared to trained athletes’ students that live at high latitudes, even if these sedentary students had a higher vitamin D food intake. Furthermore, leisure-time PA has also been associated to vitamin D levels in cancer survivor patients; however, differences between outdoor and indoor PA were found [46]. In our sample, self-report PA levels, as well as leisure PA, were not associated to vitamin D deficiency or insufficiency. A previous study showed that people who practice mountain sports are associated to a lower risk of serum vitamin D deficiency, while this association was not observed for people who practice nautical sports [47]. Moreover, a previous study of US population reported similar results, although the association was strongest to PA measure by accelerometers than by self-report [48]. Therefore, this could be a reason of non-association in our data. Our data were self-reported and does not discriminate between outdoor or indoor physical activities, therefore the method of measure of PA, outdoor/indoor factor and the different population (cancer patients versus healthy women) could explain these findings.

Hibler was one of the first in to associate ST with serum vitamin D levels. Their results support that PA is positively associated to high vitamin D levels, nonetheless they do not show associations between ST and vitamin D levels [49]. Furthermore, it was not found in association with between ST and vitamin D levels in a Brazilian sample. These contradictory findings could be due to the differences between analyzed populations, since the Brazilian study considered adolescents and participants suffering colorectal adenoma [22,49]. On the other hand, no previous studies that correlate the commute mode with vitamin D deficiency or insufficiency were found, only some activities that increase the UV-light exposition. In this sense, our results provide valuable information that shows the beneficial effect of active commuting on vitamin D levels, since the commute mode has been related positively with better health [50], low type 2 diabetes risk [51], cancer, cardiovascular disease, and all-mortality causes [52]. 

The joint association between commute mode, ST, and serum vitamin D levels has not been studied. Physical Activity time and ST were jointly evaluated in the previous Chilean national health survey, in order to estimate cardiovascular risk. The active/low sedentary behavior group presented lower cardiovascular risk factors such as hypertension, obesity, and type 2 diabetes [53]. Collectively, active commuting changes were associated to better PA patterns [54]. Therefore, both active commuting as low ST could be an important strategy to increase serum vitamin D levels and avoid the deficiency, considering the arguments exposed above where PA pattern has been estimated as a determinant variable, specifically in postmenopausal women [55].

Vitamin D deficiency is related to musculoskeletal diseases such as rickets and osteomalacia, or several infectious and metabolic processes [56]. Thus, it has been recommended the increase of vitamin D intake [57]. Indeed, in a Chilean population it has been demonstrated the combined beneficial effect of vitamin D supplementation plus exercise on vitamin D serum levels, bone density and functional capacity [58]. Despite this, two recent systematic-reviews, shows that vitamin D supplementation do not have a significant effect on fracture incidence [59] and musculoskeletal health [60]. On the other hand, aging is associated to a progressive bone mass decrease, thus remain physically active is one of the main strategies to combat this continuous loss [13,61]. An alternative to PA is the active commuting, most prevalent behavior associated to active commuting in older population is walking; especially in this population, the benefits of active commuting represents the possibility of independence and autonomy [62,63]. That allows increased sunlight exposure, and therefore, a greater possibility of vitamin D absorption. These results could have important public health implications, since several health problems are associated to low vitamin D levels. In this sense, decrease ST and increase active commuting could be useful strategies against these problems.

The mechanism implicated in this relationship remains unclear, physical activity has been related to sun exposure and vitamin D levels. Nevertheless, this report, as well as other works, has found this same association regardless of sun exposure [47,64]. Another hypothesis is related to physical activity effect and bone metabolism, suggesting an interaction between calcium and vitamin D absorption [65]. Additionally, it has been proposed a close link between sedentary time and increase adiposity, since adiposity is related to decline vitamin D levels [66,67]. Hence, in light to support these hypotheses, more researches are needed.

Important strengths in this study include the population-based sampling method and the wide consideration of potential confounders. However, this study has some limitations. The principal limitation is that cross-sectional study design does not allow to draw causal relationships as was addressed above. Thus, it is not possible to establish whether PA can lead to a vitamin D deficiency or participants with vitamin D deficiency have less PA. Another limitation is about sunlight exposure; despite having considered a question about sunlight exposure, self-report nature and dichotomy response do not grant accurate information about the real amount exposure. On the other hand, the specific vitamin D intake estimation could not be obtained from the used questionnaire and PA questionnaires utilized in our paper often fail to provide sufficient detail on activity type, frequency, duration, and intensity, especially in older adults. Finally, another limitation was the lack of calorie measures of total energy, fat and sugar intake, which could not be obtained from the questionnaire used. 

## 5. Conclusions

We found that high ST is associated with vitamin D deficiency in adult women as well as, passive commuting is associated with vitamin D deficiency. Moreover, there is a joint association of high ST/passive commuting on vitamin D deficiency and insufficiency in both groups. These novel results may add key information for public policy in Chile related to health system approach. In this sense, lifestyle recommendations are needed in order to establish specific recommendations, since the patterns of PA and ST could affect differentially vitamin D status according to age. Further research directions should establish the causal effect of PA and ST patterns, as well as establish the vitamin D deficiency implications in different pathologies in the studied Chilean population.

## Figures and Tables

**Table 1:** Descriptive characteristic of the adults and older women.

Variables, Mean ± SD	Adults Women (1245)	Older Women (686)
Age (years)	35.4 ± 8.5	73.6 ± 6.6
Body mass index (kg/m^2^)	29.2 ± 5.7	29.3 ± 5.3
Vitamin D levels (ng/mL)	20.2 ± 8.0	18 ± 8.5
Nutritional Status (*n*: %)		
Underweight	7 (0.6)	6 (0.9)
Normal weight	297 (23.9)	139 (20.3)
Overweight	455 (36.5)	245 (35.7)
Obese	486 (39.0)	296 (43.1)
Physical activity (*n*: %)		
Inactive	532 (42.7)	393 (57.3)
Active	713 (57.3)	293 (42.7)
Sedentary time (*n*: %)		
Low Sedentary time	868 (69.7)	508 (74.1)
Middle Sedentary time	260 (20.9)	138 (20.1)
High Sedentary time	117 (9.4)	40 (5.8)
Leisure-time physical activity (*n*: %)		
30 min, 3 times per week	153 (12.3)	34 (5.0)
Less than 30 min, 3 times per week	1092 (87.7)	652 (95.0)
Commute mode (*n*: %)		
Active Commuting	291 (23.4)	157 (22.9)
Passive Commuting	954 (76.6)	529 (77.1)
Educational Level (*n*: %)		
Primary	96 (7.7)	415 (60.4)
Secondary	725 (58.2)	224 (32.7)
Beyond secondary	424 (34.1)	47 (6.9)
Dairy consumption (*n*: %)		
Three times a day	84 (6.7)	46 (6.7)
Less than three times a day	79 (6.3)	55 (8)
Once a day	428 (34.4)	293 (42.7)
Every two days	223 (17.9)	124 (18.1)
At least once a week	242 (19.4)	104 (15.2)
At least once a month	73 (5.9)	22 (3.2)
Never	116 (9.3)	42 (6.1)
Menopausal status (*n*: %)		
Yes	48 (3.9)	693 (93.1)
No	1197 (96.1)	47 (6.9)
Vitamin D deficiency (<12 ng/mL) (*n*: %)		
<12 ng /mL	204 (16.4)	181 (26.4)
≥12 ng /mL	1041 (83.6)	505 (73.6)
Vitamin D insufficiency (<20 ng/mL) (*n*: %)		
<20 ng /mL	642 (51.6)	445 (64.9)
≥20 ng /mL	603 (48.4)	241 (35.1)
Sunlight exposure (*n*: %)		
Little	722 (58)	502 (73.2)
Much	523 (42)	184 (26.8)
Region (latitude *) (*n*: %)		
XV. Arica y Parinacota (−18.474)	94 (7.6)	30 (4.4)
I. Tarapacá (−20.213)	64 (5.1)	27 (3.9)
II. Antofagasta (−23.652)	57 (4.6)	23 (3.4)
III. Atacama (−27.366)	70 (5.6)	20 (2.9)
IV. Coquimbo (−29.953)	61 (4.9)	45 (6.6)
V. Valparaíso (−33.035)	125 (10)	85 (12.4)
XIII. Metropolitana (−33.456)	186 (14.9)	104 (15.2)
VI. L. Bdo. O’Higgins (−34.170)	74 (5.9)	33 (4.8)
VII. Maule (−35.426)	88 (7.1)	60 (8.7)
VIII. Bíobío (−36.826)	138 (11.1)	59 (8.6)
IX. La Araucanía (−38.739)	66 (5.3)	27 (3.9)
XIV. Los Ríos (−39.814)	56 (4.5)	48 (7)
X. Los Lagos (−41.469)	55 (4.4)	40 (5.8)
XI. Aysén (−45.575)	64 (5.1)	38 (5.5)
XII. Magallanes y Antártica (−53.154)	47 (3.8)	47 (6.9)

SD, standard deviation. * Coordinates have been calculated based on the world geodetic system (standard WGS84).

**Table 2:** Odds ratio for vitamin D deficiency according to different criteria for each physical activity pattern.

	Adults (1245)						Older (686)					
Outcome	(<12 ng/mL)			(<20 ng/mL)			(<12 ng/mL)			(<20 ng/mL)		
	OR	(95% CI)	*p*	OR	(95% CI)	*p*	OR	(95% CI)	*p*	OR	(95% CI)	*p*
*Sedentary time*												
Low sedentary time	1.0	Ref.		1.0	Ref.		1.0	Ref.		1.0	Ref.	
Middle sedentary time	2.4	1.6–3.6	**<0.001**	1.7	1.2–2.3	**0.001**	1.9	1.2–2.9	**0.004**	1.152	0.8–1.7	0.505
High sedentary time	2.6	1.6–4.3	**<0.001**	2.1	1.4–3.2	**0.001**	1.9	0.9–3.8	0.074	1.672	0.8–3.6	0.184
*Physical activity level*												
Active	1.0	Ref.		1.0	Ref.		1.0	Ref.		1.0	Ref.	
Inactive	0.9	0.7–1.3	0.6	1.0	0.8–1.3	0.795	1.2	0.8–1.7	0.393	1.2	0.9–1.7	0.207
*Leisure-time physical activity*												
30 min 3 times/week	1.0	Ref.		1.0	Ref.		1.0	Ref.		1.0	Ref.	
Less 30 min 3 times/week	1.0	0.8–1.3	0.795	1.1	0.7–1.5	0.717	1.2	0.5–2.9	0.644	1.3	0.6–2.6	0.502
*Commuting*												
Active commuting	1.0	Ref.		1.0	Ref.		1.0	Ref.		1.0	Ref.	
Passive commuting	1.1	0.7–1.6	0.755	1.5	1.2–2.0	**0.003**	1.7	1.1–2.7	**0.020**	1.7	1.1–2.4	**0.007**

OR: Odds ratio. CI: confidence interval. Results of binary regression logistic analysis adjust by age, region, dairy consumption, menopause, education level, and sunlight exposure. Significant values (<0.05) in bold.

## Supplementary Materials

The following are available online at http://www.mdpi.com/2072-6643/11/2/300/s1, Figure S1: Vitamin D levels of all survey participants along Chilean territory. Population from North of Chile exhibit higher serum vitamin D levels compared to the population from the south territory, being rather deficient for the latter. It is noteworthy, that no region’s present population hold the recommended serum vitamin D levels, which is higher than 30 ng/mL.
