# Supplementary material for: Passive Commuting and Higher Sedentary Time Is Associated with Vitamin D Deficiency in Adult and Older Women: Results from Chilean National Health Survey 2016–2017
Source: Nutrients. 2019 Jan 31;11(2):300. doi: 10.3390/nu11020300 (PMC6412538; doi:10.3390/nu11020300)

Supplementary figure legends

Figure S1. Vitamin D levels of all survey participants along Chilean territory. Population from North of Chile exhibit higher serum vitamin D levels compared to population from the south territory, being rather deficient for the latter. It is noteworthy, that no region present population with recommended serum vitamin D levels, which is higher than 30 ng/ml.

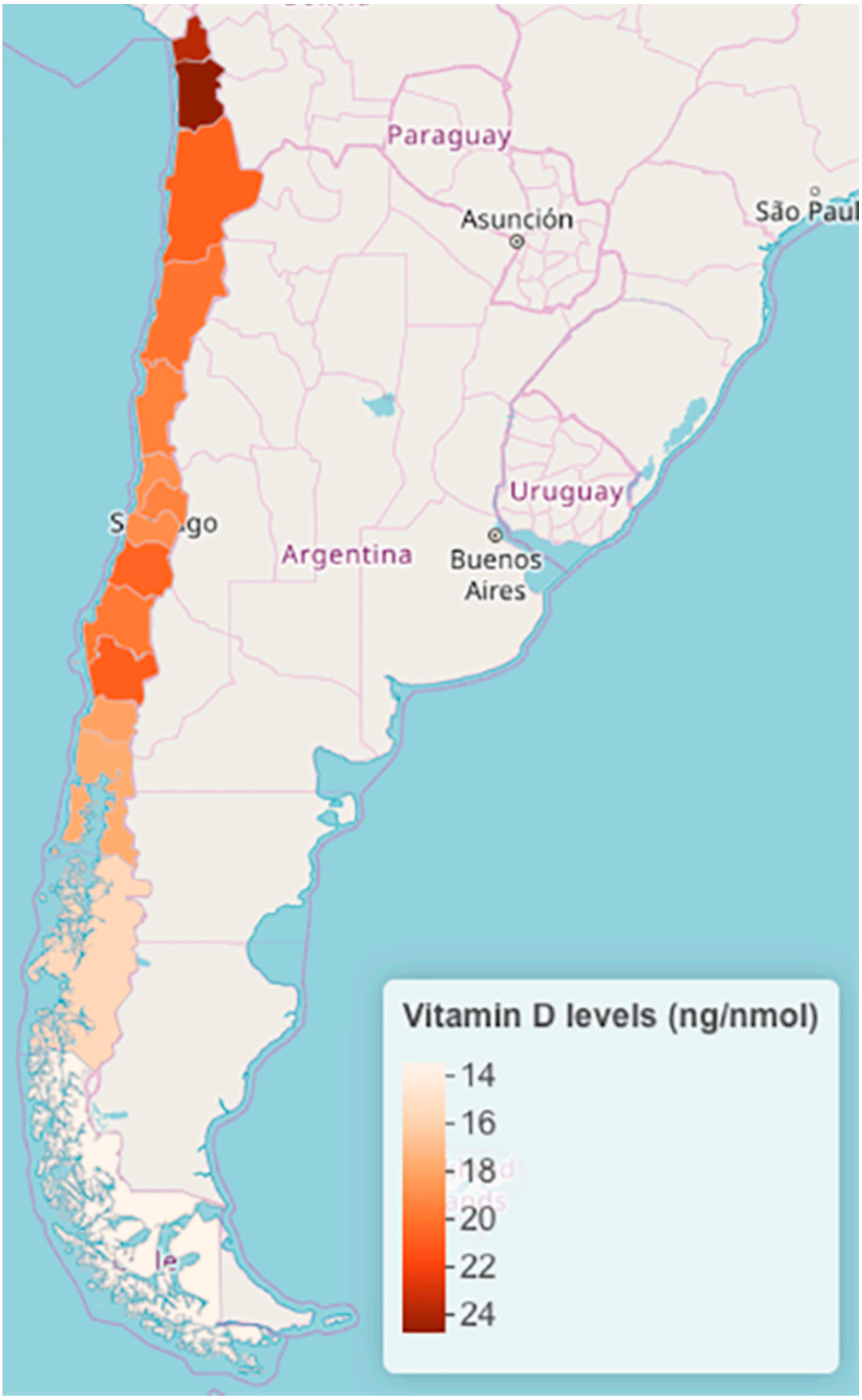

Supplement: Supplementary file 1 [file nutrients-11-00300-s001.pdf]
